# Targeting Cancer: Microenvironment and Immunotherapy Innovations

**DOI:** 10.3390/ijms252413569

**Published:** 2024-12-18

**Authors:** Irena Barbara Padzińska-Pruszyńska, Bartłomiej Taciak, Łukasz Kiraga, Anna Smolarska, Małgorzata Górczak, Paulina Kucharzewska, Małgorzata Kubiak, Jacek Szeliga, Agata Matejuk, Magdalena Król

**Affiliations:** 1 Center of Cellular Immunotherapies, Warsaw University of Life Sciences, 02-787 Warsaw, Poland; irena_pruszynska1@sggw.edu.pl (I.B.P.-P.); bartlomiej_taciak@sggw.edu.pl (B.T.); anna_smolarska@sggw.edu.pl (A.S.); malgorzata_gorczak@sggw.edu.pl (M.G.); paulina_kucharzewska-siembieda@sggw.edu.pl (P.K.); malgorzata_kubiak@sggw.edu.pl (M.K.); jacek_szeliga@sggw.edu.pl (J.S.); 2 Division of Pharmacology and Toxicology, Department of Preclinical Sciences, Institute of Veterinary Medicine, Warsaw University of Life Sciences, 02-787 Warsaw, Poland; lukasz_kiraga@sggw.edu.pl; 3 Department of Immunology, Collegium Medicum, University of Zielona Góra, 65-046 Zielona Góra, Poland; a.matejuk@inz.uz.zgora.pl

**Keywords:** cancer immunotherapy, tumor microenvironment, tumor-associated macrophages, natural killer cells, dendritic cells, Tregs

## Abstract

In 2024, the United States was projected to experience 2 million new cancer diagnoses and approximately 611,720 cancer-related deaths, reflecting a broader global trend in which cancer cases are anticipated to exceed 35 million by 2050. This increasing burden highlights ongoing challenges in cancer treatment despite significant advances that have reduced cancer mortality by 31% since 1991. Key obstacles include the disease’s inherent heterogeneity and complexity, such as treatment resistance, cancer stem cells, and the multifaceted tumor microenvironment (TME). The TME—comprising various tumor and immune cells, blood vessels, and biochemical factors—plays a crucial role in tumor growth and resistance to therapies. Recent innovations in cancer treatment, particularly in the field of immuno-oncology, have leveraged insights into TME interactions. An emerging example is the FDA-approved therapy using tumor-infiltrating lymphocytes (TILs), demonstrating the potential of cell-based approaches in solid tumors. However, TIL therapy is just one of many strategies being explored. This review provides a comprehensive overview of the emerging field of immuno-oncology, focusing on how novel therapies targeting or harnessing components of the TME could enhance treatment efficacy and address persistent challenges in cancer care.

## 1. Introduction

In 2024, it was estimated that 2 million new cancer cases would be diagnosed in the United States, with 611,720 fatalities expected from the disease [1]. Global projections indicate over 35 million new cancer cases by 2050, representing a 77% increase from the 20 million cases recorded in 2022 [2]. In the United States, the most prevalent cancers, in descending order of estimated new cases for 2024, are breast cancer, prostate cancer, lung and bronchus cancer, colon and rectum cancer, melanoma, bladder cancer, kidney and renal pelvis cancer, non-Hodgkin lymphoma, endometrial cancer, pancreatic cancer, leukemia, thyroid cancer, and liver cancer [1].

**Figure 1:**
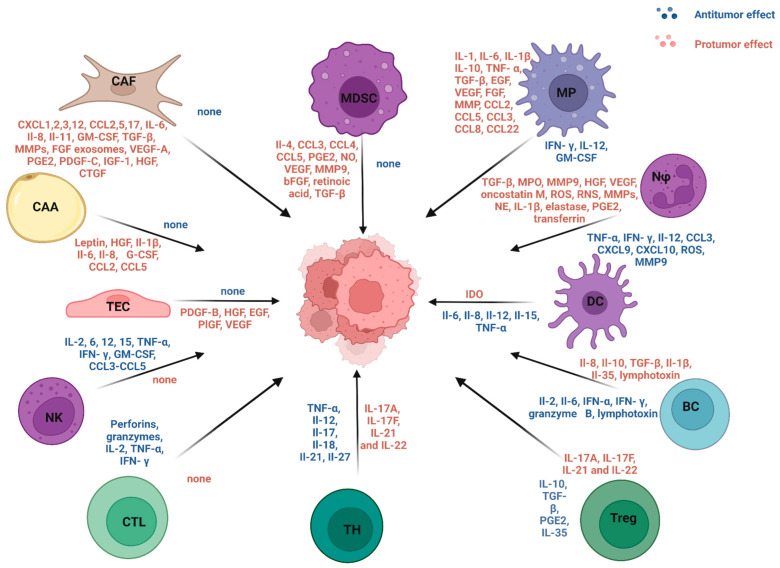
Pro- and antitumor effects of the TME cells. The tumor microenvironment consists of cells with pro-tumor properties (Myeloid-derived suppressor cells (MDSC), Cancer-associated fibroblasts (CAF), Cancer-associated adipocytes (CAA), Tumor endothelial cells (TEC)), anti-tumor properties (Natural killer cells (NK), Cytotoxic T lymphocytes (CTL)), as well as cells with mixed properties (T helper lymphocytes (TH), T regulatory lymphocytes (Treg), B lymphocytes (BC), dendritic cells (DC), neutrophils (Nφ), macrophages (MP)).

Cancer remains a leading cause of death globally, presenting significant challenges to healthcare systems worldwide [3]. The field of oncology has seen considerable advances, resulting in a 31% reduction in cancer-related mortality since 1991 [4]. This progress in cancer treatment can largely be attributed to enhanced understanding of tumor physiology and its surrounding environment, as well as the development of targeted therapies and immunotherapies that have been designed based on this knowledge. Cancer treatment presents significant challenges, primarily due to the heterogeneity of the disease. Unlike single-pathology conditions, each cancer type exhibits unique characteristics, and even tumors classified under the same type can differ significantly from person to person because of genetic mutations and variations. Crucial aspects of cancer, such as treatment resistance, the presence of cancer stem cells, drug escape mechanisms, epigenetic modifications, and metastatic behavior, underscore the complexity of this multifaceted disease, one of the deadliest globally [5]. Moreover, the tumor microenvironment plays a pivotal role, further complicating treatment strategies. This microenvironment, in concert with cancer cells, establishes a complex network of interactions that significantly influences tumor growth and metastasis. The cells found in the TME and the pro- and anti-cancer molecules they produce are shown in Figure 1. This intricate interplay underscores the need for multifaceted treatment approaches that address both the tumor and its microenvironment. The aim of this publication is to summarize the emerging unique opportunities for treating cancer by using the tumor microenvironment, rather than cancer cells directly, as the target of therapy.

## 2. The Tumor Microenvironment (TME)

The TME is a complex biological system where tumor tissue components, immune cells, blood vessels, and biochemical factors interact (Table 1). Breakthroughs in the development of novel cancer therapies have emerged from understanding the fundamental mechanisms and molecular bases of interactions within the TME. Understanding the role of individual cells and factors in tumor growth and metastasis has led to the development of new therapeutic targets and potential biomarkers, improving diagnosis, personalizing treatment, and harnessing the potential of the immune system to combat cancer [6].

Cancer immunotherapy involves treatments that stimulate the body’s own immune mechanisms to fight cancer. These treatments include specially designed drugs that either non-specifically stimulate the patient’s immune system or selectively target tumor cells. For instance, monoclonal antibodies “mark” tumor cells, signaling the immune system to eliminate them. Such therapies can achieve long-lasting immune responses and inhibit disease progression. Immunotherapy’s advantage lies in its ability to elicit a more targeted and precise response while minimizing the side effects associated with traditional treatments. Despite the promise immunotherapy holds, much remains to be understood and discovered in this field. As knowledge and technology advance, immunotherapy is expected to play an increasingly important role in the future of cancer treatment [7].

Multiple studies performed on cancer cells have revealed a number of mechanisms for their escaping immune surveillance. These include avoidance of recognition by an organism’s immune system through lack of expression of MHC (major histocompatibility complex) class I molecules, loss of antigenic epitopes, inactivation of immune cells through production of immunosuppressive factors, expression of anti-apoptotic proteins, and decreased expression of pro-apoptotic proteins [8]. Tumor cells also interact with surrounding cells to create an optimal environment for their growth and to suppress the host immune system’s activity [9,10].

**Figure 2:**
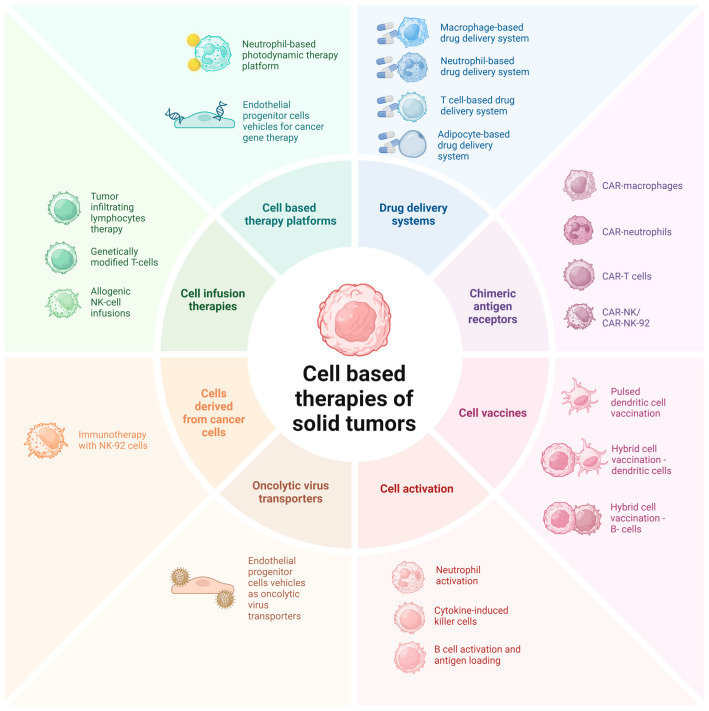
Examples of therapeutic strategies against solid tumors taking advantage of the presence of the tumor microenvironment.

This review article focuses on harnessing the cells naturally abundant in the TME, aiming to exploit their potential for innovative cancer treatments. A graphical summary of solid tumor therapies that involve TME cells is provided in Figure 2. By integrating insights from current therapies with future directions, we aim to provide a comprehensive overview of how manipulating the TME can enhance therapeutic efficacy and address ongoing challenges in cancer care.

**Table 1:** Composition of cells in TME with their functions and primary secreted factors.

Cell Type	General Function	Role in TME	Factors Through Which They Exhibit Anti-Tumor Effects	Factors Through Which They Exhibit Pro-Tumor Effects
Macrophages (MP) [11,12,13,14]	Phagocytosis of defective cells, pro-inflammatory effect, or immunomodulation	M1: Anticancer. M2: Pro-cancerous; inhibition of the inflammatory process	IFN-γ, IL-12, GM-CSF	IL-1, IL-6, IL-1β, IL-10, TNF-α, TGF-β, EGF, VEGF, FGF, MMP, CCL2, CCL5, CCL3, CCL8, CCL22
Dendritic cells (DC) [15,16]	Antigen presentation, effect on the activity of helper and regulatory lymphocytes	Disturbed antigen presentation, maturation, and infiltration of the tumor	Il-6, Il-8, Il-12, Il-15, TNF-α	IDO
(Nφ) Neutrophils [17,18,19]	Phagocytosis, ADCC, Stimulation of lymphocytes and NK cells	N1 (anti-tumor): phagocytosis, stimulation of apoptosis, activation of CD8+ T cells N2 (pro-tumor): angiogenesis, stimulation of inflammatory processes in the tumor, T cell suppression	TNF-α, IFN-γ, Il-12, CCL3, CXCL9, CXCL10, ROS, MMP9	TGF-β, MPO, MMP9, HGF, VEGF, oncostatin M, ROS, RNS, MMPs, NE, IL-1β, elastase, PGE2, transferrin
Cytotoxic T lymphocytes (CTL) [20,21]	Cytotoxic effect, stimulation of other immune cells	Disruption of cytotoxic effects by binding to PD-L1	Perforins, granzymes, IL-2, TNF-α, IFN-γ	none
T helper lymphocytes (Th cells) [22,23]	Stimulate by Th1 of dendritic cells and NK cells	Th1—inhibition of Treg, Th2 and M2 by Il-10, Il-4, TGF-β Th2—stimulation of M2 and inhibition of Th1	TNF-α, Il-12, Il-17, Il-18, Il-21, Il-27	IL-17A, IL-17F, IL-21 and IL-22
T regulatory lymphocytes (Treg) [24,25]	Protection against autoimmune reactions	Immunosuppression, facilitating of immune tolerance	IL-10, TGF-β, PGE2, IL-35	TGF-β, IL-2, IL-10, IL-35
B lymphocytes (BC—B cell) [26,27,28]	Antibody production, complement activation, antigen presentation	Stimulation of Treg, enhanced angiogenesis, inhibition of Tc, production of anti-inflammatory factors	Il-2, Il-6, IFN-α, IFN-γ, granzyme B, lymphotoxin	Il-8, Il-10, TGF-β, Il-1β, Il-35, lymphotoxin
Natural Killer cells (NK) [29,30]	Cytotoxic effects, stimulation of T lymphocytes and dendritic cells	Inhibition of NK activity by disrupting their activation	IL-2, 6, 12, 15, TNF-α, IFN-γ, GM-CSF, CCL3-CCL5	none
Cancer-associated fibroblasts (CAF) [31,32,33]	none	Pro-inflammatory effects, secretion of growth factors, angiogenesis	none	CXCL1,2,3,12, CCL2,5,17, IL-6, Il-8, Il-11, GM-CSF, TGF-β, MMPs, FGF exosomes, VEGF-A, PGE2, PDGF-C, IGF-1, HGF, CTGF
Cancer-associated adipocytes (CAA) [34,35,36]	none	Secretion of adipokines	none	Leptin, HGF, Il-1β, Il-6, Il-8, G-CSF, CCL2, CCL5
Myeloid-derived suppressor cells (MDSC) [37,38]	none	Suppression of the immune response, promotion of angiogenesis	none	Il-4, CCL3, CCL4, CCL5, PGE2, NO, VEGF, MMP9, bFGF, retinoic acid, TGF-β
Tumor endothelial cells [39,40,41]	none	Source of CAFs, attenuation of Tc immune cell response	none	PDGF-B, HGF, EGF, PlGF, VEGF

### 2.1. T Lymphocytes

Cytotoxic T lymphocytes (CTLs), or CD8+ T cells, are crucial for combating infections and cancer. They recognize specific antigens presented by MHC-1 molecules on antigen-presenting cells and, upon activation, release cytolytic mediators like granzyme and perforin to induce apoptosis in target cells. They also secrete cytokines such as IFN-γ and TNF-α to enhance immune responses [42,43,44].

In the tumor microenvironment (TME), the presence and activity of CTLs are key indicators of prognosis. Tumors with high CTL infiltration and PD-L1 expression are classified as “hot” and typically respond well to immunotherapy, whereas “cold” tumors have sparse CTL presence and lower antigen presentation, making them less responsive to treatment [43,45,46,47]. Despite their presence, CTLs can be hindered by the TME’s immunosuppressive environment, which includes factors like perforin-degrading enzymes and down-regulated MHC-1 expression. The TME can also trap CTLs in a dense extracellular matrix, impairing their ability to kill cancer cells. Tumors exploit immune checkpoint pathways, leading to reduced function and proliferative capacity, which leads to CTL exhaustion.

CD4+ T-helper (Th) cells are central to adaptive immunity, orchestrating both pathogen and tumor responses through cytokine production. They regulate other immune cells, supporting cytotoxic CD8+ T cells, B cell antibody production, macrophage activation, and dendritic cell maturation. Naïve CD4+ T cells can differentiate into Th1, Th2, Th17, Tfh, and Treg subsets, each with distinct roles in the TME [48,49]. In early tumor progression, Th cells infiltrate the TME, and their presence often correlates with a better prognosis in many solid tumors [50]. However, tumors can eventually evade immune responses, leading to progression and metastasis. CD4+ Th cells are of growing interest in immunotherapy, but their high plasticity and ability to switch phenotypes can complicate their role in tumor immunity. For instance, Tregs can convert to Th1 or Th17 types, while Th1 cells secrete cytokines like IFN-γ and IL-2 that enhance anti-tumor immunity, and Th2 cells release IL-4 and IL-10, which can promote tumor growth [51,52,53]. Th1 cells support anti-tumor responses by recruiting CD8+ T cells and activating dendritic cells, while Th2 cells can contribute to tumor progression through M2 macrophage polarization and the accumulation of myeloid-derived suppressor cells. Th17 cells have dual roles, showing both pro- and anti-tumor effects, depending on the cancer type. T follicular helper (Tfh) cells aid in B cell antibody production and enhance anti-tumor responses through IL-21 [54,55,56].

#### T Cell Cancer Therapies

Lymphocytes are the major group of cells targeted or harnessed in anti-cancer treatment, from modulating their responses to enhance anti-tumor effects by balancing Th1/Th2 and Th17/Treg interactions [57], through TIL therapies and breakthrough check-point inhibitor (CPI) immunotherapies, to advanced CAR-T cell constructs. The advances in the area of therapies targeting immune checkpoints (ICIs), such as cytotoxic T lymphocyte antigen 4 (CTLA-4) and programmed death receptor 1 (PD-1) with its ligand PD-L1, have opened the door to revolutionary therapeutic strategies. Current immunotherapy efforts focus on reactivating exhausted CTLs using checkpoint inhibitors such as antibodies against CTLA-4, PD-1, and PD-L1. Combining these therapies with standard treatments can enhance their effectiveness and restore CTL anti-tumor activity [58,59]. The first FDA-approved drug to block immune checkpoints was the human anti-CTLA-4 monoclonal antibody, ipilimumab [59], used for the treatment of melanoma (CTLA-4—a co-inhibitor that inhibits T cell activation). Further drugs targeting immunological checkpoints are anti-PD-1 antibodies (pembrolizumab and nivolumab), which feature a higher safety profile than anti-CTLA-4 [60]. PD-1 and its ligand PD-L1 are co-inhibitory molecules that regulate the response of T lymphocytes on the surface of tumor cells [61]. Tumor cells expressing the PD-L1 ligand bind to the PD-1 receptor on the surface of lymphocytes, leading to the inhibition of their cytotoxicity against tumor cells [62]. By using anti-PD-1 or anti-PD-L1 antibodies, the lymphocyte response is not dumbed down.

While immune checkpoint inhibitors (ICIs) have significantly advanced cancer care, issues like inadequate antitumor T cell responses and impaired memory formation often hinder their effectiveness. Cell-based therapies are emerging as transformative treatments for solid tumors, addressing limitations faced by immune checkpoint inhibitors, such as resistance and insufficient T cell activity. Adoptive cellular therapy (ACT) offers a promising solution by enhancing T cells’ number, specificity, and functionality against tumor cells [63]. This approach, which has already revolutionized the treatment of hematologic cancers, is now being explored for solid tumors through three primary modalities: tumor-infiltrating lymphocytes (TILs), genetically engineered T cell receptors (TCRs), and chimeric antigen receptor (CAR)-T cells. TIL therapy expands naturally occurring T cells from tumors, while TCRs and CAR-T cells involve genetic modifications to target specific tumor antigens. Despite its early stages in solid tumor treatment, ACT holds immense potential to overcome current therapeutic barriers and pave the way for more effective cancer treatments [64].

The latest innovation in the cell therapy field, approved by the FDA, uses tumor-infiltrating lymphocytes (TILs) from a patient’s own tumor. These TILs are cultivated to billions in the lab and reintroduced to the patient, aiming to harness and amplify the body’s natural cancer-fighting capabilities [65]. Stanford Medicine recently became the first center to treat a patient with metastatic melanoma using this new FDA-approved cell-based therapy. This therapy, commercially known as Amtagvi (lifileucel), provides new hope for patients with advanced melanoma who have not responded to existing treatments [66]. Clinical trials have shown that about 30% of patients experienced tumor shrinkage or disappearance, with 40% showing no cancer progression for at least 18 months after treatment. The FDA’s approval of lifileucel represents a significant breakthrough, expanding cell-based therapies into the realm of solid tumors and offering a new standard of care for patients with advanced melanoma.

To increase the affinity of T lymphocytes to tumors, genetic engineering has been employed to create chimeric antigen receptors (CARs). These genetically modified receptors [67] provide specific immune properties to the effector cell. Originally modified CAR-expressing T cells have been engineered to selectively recognize structures on the surface of target (tumor) cells, independent of the MHC protein complex. CARs have three domains. The first is an extracellular domain involved in antigen recognition, forming a single-chain antibody fragment (scFv). Next is the trans-membrane domain, which anchors CAR in the cell and is responsible for its stability and interaction with other membrane proteins. The third is intracellular (signaling), stimulating T cells to proliferate, cytolyze, and secrete cytokines to eliminate target cells. Several generations of CARs already exist, and each successive generation differs in its intracellular domain. The first generation of CARs contains a CD3ζ signaling domain. The activity of T cell receptors belonging to this generation, however, is limited because activation of T cells requires signaling from the CD3 and CD28 complex. The second generation of CARs involves the addition of a costimulatory domain: CD28 or 4-1BB. In contrast, third-generation CARs on top of the intracellular domain CD3ζ contain two costimulatory domains, both CD28 and 4-1BB [68]. Fourth-generation CAR-T cells (T cells redirected for universal cytokine killing, TRUCK) exhibit more effective anti-tumor activity by releasing cytokines, antibodies (e.g., anti-PD-1) and enzymes that can degrade the extracellular matrix in solid tumors. An effect of the use of TRUCK is the release of IL-12 upon antigen recognition. IL-12 stimulates T cells and increases the secretion of IFN-γ in the tumor microenvironment [67]. The use of CAR-T therapies in the treatment of hematologic malignancies has achieved high efficacy. Since 2017, the FDA has approved CAR-T therapies in acute lymphocytic leukemia, multiple myeloma, and various types of lymphoma. In the pivotal ELIANA trial (ClinicalTrials.gov: NCT02435849) conducted on pediatric and young adult patients with relapsed or refractory acute lymphoblastic leukemia, an overall response rate (ORR) of 82% was observed, encompassing both complete remission (CR) and complete remission with incomplete count recovery (CRi). The latest analysis of this study indicates that the 5-year relapse-free survival rate among patients who initially achieved CR or CRi following administration of tisagenlecleucel is 49% [69]. Tecartus (brexucabtagene autoleucel) has shown promising results in treating mantle cell lymphoma (MCL). According to the pivotal ZUMA-2 trial, Tecartus demonstrated a high ORR of 91%, with 68% of patients achieving a complete response. The median duration of response was 28.2 months, and the median overall survival among treated patients was 46.6 months. These results are notable for their durability and efficacy in a patient population with relapsed or refractory MCL [70]. However, the implementation of this technology in solid tumors faces many hurdles, and targeting cells to the deeper regions of the tumor is particularly problematic.

T cell receptor (TCR)-based adoptive therapy involves the ex vivo genetic modification of lymphocytes to target specific tumor-associated antigens [71]. These genetically engineered lymphocytes express TCRs that are specific for cancer antigens. Unlike chimeric antigen receptors (CARs), TCRs have the unique ability to recognize peptides presented by human leukocyte antigen (HLA) molecules, which are derived from proteins originating in all cellular compartments of the target cell. The genetic modification of T cell receptors is typically achieved through transduction using viral vectors [72]. This adoptive T cell therapy was tested in patients with hematologic cancers, melanoma, and cervical carcinoma, with satisfactory results [73,74,75]. One notable example is the FDA-approved afamitresgene autoleucel (afami-cel), tested in patients with synovial sarcoma. In this pivotal trial, 44 participants received the therapy, resulting in a 39% objective response rate, including complete responses in two patients. The study reported manageable side effects, primarily cytokine release syndrome, which was mostly mild [76]. Due to their ability to recognize a broader range of tumor antigens, many researchers posit that TCRs may offer superior efficacy in treating solid tumors compared to CAR-T therapies [77].

Unfortunately, T cell therapies have their limitations and are burdened with the risk of adverse effects. The most common problem associated with CAR-T cell therapy is cytokine release syndrome (CRS). It results from the rapid release of cytokines from immune cells in response to modified CAR-T lymphocytes [78]. Another well-described side effect of CAR-T cell infusion is the occurrence of immune cell–associated neurologic syndrome (ICANS). It occurs most often in patients who also have CRS. Its clinical symptoms also result from excessive production of certain proinflammatory cytokines [79]. Unlike side effects associated with chemotherapy, generally toxicity associated with targeted therapies is temporary and resolves when the CAR-T cells finish expanding, or are eradicated or exhausted [73]. Since almost 50% of patients in early treatment trials receiving CAR-T therapy required intensive care management, it seems obvious that managing side effects is as important for targeted therapies as it is for traditional cancer therapies [79,80].

### 2.2. Regulatory T Lymphocytes

Regulatory T lymphocytes (Tregs) are responsible for suppressing the immune response against foreign antigens as well as autoantigens, contributing to the maintenance of immune tolerance [81]. The basic division of regulatory lymphocytes distinguishes between natural tTreg (thymus-derived Treg cells) and induced pTreg (peripherally derived Treg cells). They differ in the level of expression of certain transcription factors, such as helios, which are actively involved in immunosuppression (tTreg shows its overexpression, while pTreg often does not show it at all) [82]. Both subpopulations can show increased suppression when activated as effector regulatory cells—eTreg [83]. In recent years, IL-35-induced lymphocytes have also begun to be included in these subpopulations [84].

#### Therapies Utilizing Tregs

The ability of regulatory T cells to suppress the immune response against antigens, both foreign and autoantigens, makes them an attractive target for therapy in various autoimmune diseases, allergies, and transplantation. In recent years, several promising Treg cell-based therapeutic strategies have been developed that have the potential to change the frontiers of immune disease treatment. The first of these strategies is autologous Treg transfusion-based therapy. It involves isolating Treg lymphocytes from the patient, multiplying them in vitro, and then transfusing them back into the patient’s body [85]. This approach increases the number of functional Tregs, which can effectively suppress autoimmune reactions and excessive immune responses. Another strategy is to stimulate Tregs in vivo. Through the use of substances such as cytokines (e.g., IL-2), Treg populations existing in the body can be activated, leading to improved control of immune responses [86]. This approach obviously obviates the need for in vitro cell expansion and transfusions.

### 2.3. Macrophages

Macrophages in the tumor microenvironment (TME) play critical roles in tumor progression and immune response regulation. They originate from tissue-resident macrophages (TRMs) and circulating monocytes, exhibiting high plasticity. An extremely important feature of monocytes and macrophages is their ability to migrate to the inflammatory sites to eliminate the cause of inflammation and initiate the tissue healing and repair process [23,87]. Macrophages require initiating factors for effective migration. The first of these is pathogen-associated molecular patterns (PAMPs), which come from pathogens attacking the body. The second is damage-associated molecular patterns (DAMPs), released from dead or damaged cells. Additionally, macrophages migrate to tissues through chemotaxis in response to chemokines and cytokines secreted by activated T lymphocytes. Macrophages possess polarizing abilities and can reorganize their cytoskeleton, facilitating their effective migration [88]. They migrate more quickly than other leukocytes (neutrophils, T lymphocytes) but more slowly than mesenchymal cells (e.g., fibroblasts) [89].

Macrophages can adopt different phenotypes, notably M1 and M2, influenced by local cytokines [90,91]. M1 macrophages, activated by IFN-γ and LPS, promote pro-inflammatory responses, secrete cytokines like TNF-α and IL-12, and are involved in pathogen phagocytosis and tumor cell elimination [92]. In contrast, M2 macrophages, induced by IL-4 and IL-13, support immunosuppression, tissue remodeling, angiogenesis, and tumorigenesis, secreting anti-inflammatory cytokines like IL-10 and TGF-β [93,94].

#### 2.3.1. Targeting TAMs

Recent advances in cancer therapy have highlighted the potential of targeting macrophages within the tumor microenvironment (TME) [94]. Macrophages, which play a crucial role in regulating immune responses and maintaining tissue homeostasis, can significantly influence tumor progression and the efficacy of anti-cancer treatments. Various therapeutic strategies are being explored to manipulate these cells to enhance anti-tumor activity. One promising approach involves reprogramming tumor-associated macrophages (TAMs) to shift from a pro-tumorigenic (M2) phenotype to an anti-tumorigenic (M1) phenotype. This can be achieved using small molecule inhibitors, antibodies, and cytokines that modulate macrophage activation and polarization. For instance, blocking the colony-stimulating factor 1 receptor (CSF1R) pathway, which is essential for the survival and maintenance of TAMs, has been shown to reduce the population of pro-tumor macrophages and enhance anti-tumor immune responses. Additionally, therapies targeting immune checkpoints are being investigated for their ability to modulate macrophage activity in the TME [12].

#### 2.3.2. Macrophage-Based Cell Therapies

Macrophage-based cell therapies are gaining traction as a viable alternative to T cell-based therapies, particularly for solid tumors where T cell infiltration is limited. Unlike T cells, macrophages, especially tumor-associated macrophages (TAMs), are constantly replenished by circulating monocytes, which naturally home in on tumor sites. This unique ability makes them promising candidates for delivering therapeutic agents directly to the tumor microenvironment (TME). Researchers have explored various strategies utilizing macrophages’ natural tumor-homing abilities. For instance, monocytes loaded with drug nanoparticles and injected into tumor-bearing mice have demonstrated superior tumor-targeting efficiency compared to free nanoparticles [95]. Another innovative approach involves transducing monocytes with genes encoding immune-stimulatory cytokines. In one study, monocytes engineered to express IFNα under the Tie2 promoter effectively migrated to tumors, where they activated immune cells and inhibited tumor growth and angiogenesis [96]. One innovative approach involves the use of “backpacks”—soft particles containing the cytokine IFNα attached to the surface of macrophages. This strategy has been demonstrated to induce an M1 phenotype in macrophages, which is associated with anti-tumor activity. When these backpack-loaded macrophages were injected directly into tumors, they retained their M1 phenotype despite the typically immunosuppressive TME, leading to significant reductions in tumor growth and metastatic burden in mouse models [97].

In another study focusing on a murine sarcoma model, researchers identified premetastatic niches characterized by immune suppression, particularly involving myeloid cells. By genetically engineering bone marrow-derived myeloid cells to express IL-12, they were able to stimulate a type 1 immune response in the lungs. The adoptive transfer of these IL-12-expressing cells resulted in decreased metastasis and primary tumor growth, highlighting the potential of engineered macrophages to reprogram the immune environment and inhibit cancer progression [98]. During the last FOCIS conference (2024), a novel macrophage approach was presented [99], namely, loading macrophages with ferritin-drug complexes, conceptualized as Macrophage–Drug Conjugates (MDCs). Ferritin’s protein-cage structure makes it an excellent drug carrier, and macrophages can internalize substantial amounts of ferritin. A groundbreaking discovery within this study is the “TRAnsfer of Iron-binding protein” (TRAIN) process, wherein drug-loaded macrophages transfer ferritin to adjacent cancer cells through direct cell–cell contact and the formation of an immune synapse-like structure [100]. Macrophages loaded with ferritin conjugated to cytotoxic drugs exhibited potent anti-cancer activity across various orthotopic solid tumor models, including glioblastoma [101], ovarian cancer, and pancreatic cancer, leading to complete tumor elimination in these models. Additionally, these macrophages not just phagocytose cancer cells, but also activate the immune system and establish immune memory, contributing to long-term anti-tumor resistance. This pioneering work sets the stage for translating this powerful adoptive cell therapy into clinical trials, offering a promising avenue for the treatment of solid tumors.

Moreover, macrophages can be armed with engineered receptors, such as chimeric antigen receptors (CARs), to enhance their anti-tumor activity. CAR-macrophages (CAR-M) targeting HER2 have been developed to combat solid tumors. These CAR-M cells demonstrate stable M1 phenotypic functions, including phagocytosis, and can traffic to primary and metastatic tumor sites [102]. The first clinical data on genetically engineered macrophages in humans demonstrated that CAR-M therapies could be effective against solid tumors. The FDA has granted Fast Track designation to CT-0508 for further evaluation in clinical trials, underscoring the therapy’s potential. Current trials are ongoing at institutions like the University of Pennsylvania, University of North Carolina, and City of Hope, aiming to assess the efficacy and safety of these innovative therapies [102].

These approaches collectively underscore the potential of macrophage-based therapies in providing a robust, targeted response against various cancers, paving the way for new clinical applications and improved patient outcomes.

### 2.4. Dendritic Cells

Dendritic cells (DCs) are known as the most effective antigen-presenting cells (APCs), which can take up, transform, and present various types of antigens, including tumor antigens. They have the unique ability to stimulate naive T cells. DCs also play an important role in the innate and acquired immune response. The environment surrounding dendritic cells can lead to changes in their function, directing their activation toward classical DCs (cDCs; conventional DCs), or toward plasmacytoid DCs (pDCs), a subpopulation with tolerogenic and immunosuppressive properties [103]. Several different molecules and signaling pathways have been described that may be involved in the induction of the tolerogenic and immunosuppressive properties of tumor-associated DCs, including production of IL-10 and TGF-β; expression of IDO, iNOS and arginase: or expression of inhibitory B7-related molecules [104,105]. The tumor microenvironment can also disrupt the maturation of dendritic cells. Some subsets of immature DCs cannot provide T cells with appropriate costimulatory and cytokine signals and can induce tolerance by reducing proliferation and disrupting the function of CD4+ and CD8+ T cells. Tolerogenic DCs can produce TGF-β, which stimulates proliferation of regulatory T cells [106,107,108].

Dendritic cells have indirect and direct cytotoxic effects. Their key function in the case of cancer is the ability to present antigens to T lymphocytes and lead to the proliferation of immature T lymphocytes. Typically, antigen presentation occurs in lymph nodes; however, studies conducted in recent years have shown that antigen presentation by DCs also occurs in tumor cells [109]. Dendritic cells are used in anticancer therapies in two ways.

#### DC-Based Cancer Cell Therapies

The first DC vaccines were developed for melanoma patients. Later, vaccine studies were expanded to include lymphoma and acute myeloid leukemia, followed by myeloma and prostate cancer [110,111]. DCs are used to produce vaccines using hematopoietic progenitor myeloid cells (CD34+) isolated from the patient’s peripheral blood, as well as monocytes (CD14+), which undergo differentiation into dendritic cells. Immature DCs, during incubation, are stimulated with IL-4 and granulocyte-macrophage colony-stimulating factor (GM-CSF), among others, which cause dendritic cells to mature, or are transfected with genetic material that contains genes encoding tumor-associated antigens. Some researchers use hybrids made from the combination of dendritic cells with tumor cells [110,112]. In 2010, the DC-based Sipuleucel-T vaccine was registered and approved for use in the treatment of prostate cancer patients with the consent of the US Food and Drug Administration (FDA) [112,113].

Unfortunately, there is no evidence of the DC vaccine efficacy in clinical trials. This is primarily related to immunoregulatory compounds that are found in the tumor microenvironment, and which can also be produced by the DCs themselves. One such factor may be indoleamine 2,3-dioxygenase (IDO), which exhibits inhibitory effects on T cell activity and proliferation, and can lead to tumor growth and metastasis, as well as a reduction in the efficacy of the administered vaccine [114]. IDO (or IDO1) is the enzyme which catabolizes tryptophan and may contribute to tryptophan deficiency, and thus indirectly contributes to the inhibition of T-lymphocyte activity [115]. Zhuang et al., in their study, developed a system of delivery siRNA directed against IDO synthesis in dendritic cells. GNR gold nanowires were used as the carrier for the siRNA. The researchers demonstrated that this method (man-GNR-siIDO) can effectively silence the IDO gene in dendritic cells both in vitro and in vivo, and thus improve the efficacy of vaccine-mediated treatment [114]. Monoclonal antibodies (mAb) directed toward dendritic cell surface markers are used for therapeutic purpose to enhance DCs activity. This approach is used in both monotherapy and in combination therapies [116].

### 2.5. Natural Killer (NK) Cells

Human NK cells have been characterized based on the presence of CD56 and NKp46 markers, as well as the absence of B and T lymphocyte receptors. NK cells exert a thoroughly documented anti-tumor effect and are one of the first lines of defense against tumorigenesis. They play a major role in limiting the metastasis process and are more often found in blood vessels than in the tumor mass itself [117,118,119,120,121]. However, their action depends not only on their number, but also on the level of their activation [122,123]. The anticancer effects of natural killer cells are exerted mainly through their cytotoxic abilities, such as direct lysis occurring via perforins and granzymes, induction of apoptosis by FasL/Fas and TRAIL/TRAIL, and the release of the cytokines CCL3-5, IFN-γ, and TNF-α [124,125]. The production of these cytokines enables another, helper, action of NK cells, as it stimulates the generation of DC1 dendritic cell populations and supports the phagocytic and lytic abilities of macrophages [126]. NK cells, due to their high heterogeneity and tissue specificity, in addition to their killing and helper properties, can also perform a memory or memory-like function [30]. Performing this role requires prior exposure to specific haptens, viral infection, or cytokines such as IL-12, 15, and 18 [127,128,129]. Tumors tend to avoid activation of natural killers by decreasing activator signals and increasing inhibitory signaling for NK cells. In addition to tumor cells, myeloid-derived suppressor cells (MDSCs), tumor-associated macrophages (TAMs), and regulatory T cells (Tregs) also have the ability to inhibit natural killers by producing molecules such as Il-10, TGF-β, ADO, and IDO [130,131]. Tissue barriers surrounding a tumor mass can also act as an obstacle to NK cells, impeding tumor penetration [132].

#### 2.5.1. Targeting NK Cells

In designing NK cell therapies, researchers have leveraged key insights into NK cell biology to enhance therapeutic efficacy. The activity and efficacy of NK cells used in immunotherapy is enhanced by incubating them with cytokines or antibodies, as well as by inserting chimeric receptors on them [133]. The effector function of NK cells can be enhanced by the type I IFN and interleukins (IL-12, IL-18, and IL-15) [7]. IL-2 stimulates NK cell proliferation and enhances their cytotoxicity [134]. Lim et al. proved that allogeneic NK cells cultured with various nutrients and the cytokines IL-12, IL-15, and IL-18 have greater efficacy in treating hematologic malignancies and solid tumors [135]. Another approach is to use monoclonal antibodies that redirect NK cell cytotoxicity to target cells. Immunoglobulin G (IgG) binds to both the target cell and the CD16A receptor (FcγRIIIA, or immunoglobulin gamma Fc region receptor III-A) located on the NK cell [7]. The Fc receptor (FcR) is among the potent activators of NK cell activity. This interaction induces antibody-dependent cellular cytotoxicity (ADCC). Once activated, metalloproteinase-17 (ADAM17, a disintegrin, and metalloprotease 17) cuts the receptor from the NK cell surface, resulting in increased cytokine production and enhanced cytotoxicity directed against the tumor [133,136,137].

#### 2.5.2. NK Cell-Based Therapies

Currently developed NK cell-based therapies are of various types: autologous, allogeneic, peripheral blood-derived, stem cell-derived, or cell-line. For patient safety, cells must be irradiated before administration, which limits their effectiveness; however, they still have a high degree of toxicity [138,139]. There is also a strategy of genetically modifying NK cells into a chimeric antigen receptor (CAR), which enables better interaction of the NK cell with the tumor cell [140]. Preclinical studies testing the potential of CAR-NK in the treatment of solid tumors and hematological malignancies are currently in progress [141]. The advantages of CAR-NK therapy include easy isolation of NK cells from the patient’s blood—and therefore, relatively low cost of the therapy—and, most importantly, the low probability of producing a cytokine storm, as well as graft-versus-host disease (GVHD), as compared with CAR-T therapy [133]. Given the characteristics of NK cells, such as cytolytic properties, natural infiltration of tumor tissues may become a promising therapeutic approach in the treatment of solid tumors in clinical practice. An example is the results of recent phase I/II clinical trials of allogeneic cells in pancreatic adenocarcinoma and solid tumors with ROBO-1 expression (NCT03940820, NCT03941457, NCT03931720) [142,143,144]. NK cell-based therapies have been tested across various cancer types, including leukemia, lymphoma, and solid tumors like lung and head and neck cancers. The results have generally shown that NK cells can mediate substantial anti-tumor effects, often leading to tumor regression or stable disease. The feasibility and safety of these therapies have been confirmed in multiple phase 1 and phase 2 trials, paving the way for further research and development [145]. One limitation of the NK cell therapies may be inefficient migration to the tumor [146]. Moreover, expansion of pure NK cells population in cancer patients is challenging [7,147].

### 2.6. Neutrophils

Neutrophils (Nφ) are the most common type of non-specific immune cells and are the first cells to reach sites of developing inflammation. In an animal model of zebrafish, Il-8 has been shown to recruit neutrophils via CXCR1/CXCR2 receptors to both damaged tissues and tumor masses [148,149]. In the tumor microenvironment, they are distinguished as two subgroups, which can have either an anti-tumor (N1 neutrophils) or a pro-tumor (N2 neutrophils) function [150]. The activity of type 1 interferons induced the production of N1-type neutrophils, which are characterized by enhanced adhesion, phagocytosis, degranulation, transmigration, and improved release of neutrophil extracellular traps (NETs), in a process known as NETosis. This group is characterized by its immunostimulatory profile (i.e., TNFαhigh, CCL3high, ICAM-1high, Arginaselow, and CD-177 overexpression) [151,152,153]. On the other hand, transforming growth factor β (TGF-β) produced by tumor cells polarizes neutrophils to an N2 phenotype, which is distinguished by increased expression of Olfactomedin 4 (OLFM4) and the chemokines CCL2, 3, 4, 8, 12, and 17, and CXCL1, 2, 8, and 16 [150,153,154]. N1 neutrophils can directly kill cancer cells by releasing reactive oxygen species (ROS) and reactive nitrogen species (RNS) [155,156]. They can also promote T cell activation and recruit pro-inflammatory M1 macrophages through the production of lactoferritin [157,158]. N2 neutrophils exert their pro-tumor effects through the release of matrix metalloproteinase 9 (MMP9), which promotes angiogenesis and tumor cell migration [159,160]. Stimulation of cancer metastases may also occur through the production of the leukotriene-producing enzyme, arachidonate 5-lipoxygenase (Alox5), and transferrin. Pro-tumor neutrophils can also suppress the function of natural killer cells and CD8 T cells and recruit anti-inflammatory M2 macrophages and regulatory T cells to the tumor [161,162]. There are studies assessing the possibility of activating neutrophils using a cocktail consisting of tumor necrosis factor, CD40 agonist, and tumor-binding antibody. Studies on mice and human cells in vitro confirmed the effectiveness of this therapeutic approach [163]. In studies of melanoma and lung cancer, the activation of tumor-associated neutrophils was correlated with better responses to immunotherapies, including T cell therapies and immune checkpoint inhibitors. Patients who had an expanded neutrophil response following treatment exhibited improved tumor control and overall survival, suggesting that neutrophils could be leveraged to enhance therapeutic efficacy [164,165]. Another strategy for utilizing neutrophils in cancer therapy involves employing them as carriers for anticancer drugs. Neutrophils, akin to macrophages, exhibit chemotactic migration in response to chemokines secreted during inflammatory processes. Given that inflammation is commonly associated with many solid tumors, neutrophils serve as effective carriers for nanoparticles loaded with anticancer agents. This approach has been validated in both in vitro and in vivo studies, including murine models [166,167]. Neutrophils can also function as carriers for photosensitizing agents targeted at tumor sites. Photodynamic and photothermal therapies, which leverage ROS generated by these photosensitizers, offer a potent adjunct to the treatment of solid tumors [168].

### 2.7. B Lymphocytes

There are three main types of B lymphocytes that can be found in the TME: antigen-presenting, antibody-secreting, and regulatory (Breg). However, as with other cells in the immune system, a specific tumor microenvironment can induce the formation of a subgroup of pro-tumor B lymphocytes. Such pro-tumor cells produce antibodies directed against non-mutated proteins of their own body, as in autoimmune diseases [169]. These antibodies have the ability to form circulating immune complexes (CICs). The presence of these complexes correlates with a worse prognosis due to the activation of the Fcγ receptors of macrophages and mast cells and their pro-angiogenic effects [170,171,172]. Tumor cells, but also immune cells located in the TME, produce cytokines whose interaction with B lymphocytes is responsible for the expansion, or differentiation of a specific heterogeneous subgroup of pro-neoplastic lymphocytes called Breg [173,174,175,176]. Breg lymphocytes found in the TME can produce compounds with immunosuppressive properties, mainly IL-10, but also IL-35 and TGF-β [177,178]. This group of lymphocytes can also stimulate chronic inflammation, and thus tumor growth, and can inhibit the response of CD4+ T cells and NK cells [169,179,180]. B lymphocytes can be modified both in vivo and ex vivo by introducing specific tumor antigens, thereby facilitating the activation of T lymphocytes and enhancing their antitumor response [181,182,183]. Tumor-specific antigens can be introduced into B cells through various methods, including peptide loading of tumor antigens [184], RNA or DNA transfection [185], electroporation [182], and transduction using viral vectors [186]. The ex vivo modification of B lymphocytes offers the additional advantage of enabling their concurrent activation, which can, for example, prevent reversion towards a pro-tumor phenotype [187,188]. Another approach to harness the anticancer potential of B lymphocytes involves developing vaccines comprising a fusion of peripheral blood B lymphocytes from healthy donors with autologous tumor cells [189]. This strategy exploits the antigenicity of tumor cells in combination with the immunogenic properties of B lymphocytes expressing MHC class II molecules on their surface. A study at Northwestern University investigated a B cell vaccine (BVax) that activates B cells to produce antibodies specifically targeting glioblastoma, a particularly aggressive cancer. This approach has shown promise in preclinical models, where B cells were able to infiltrate the tumor, produce antibodies, and activate tumor-killing T cells [190]. While clinical trials are still in the early stages, B cell activation therapies hold significant potential for advancing cancer immunotherapy, especially by leveraging their ability to both directly attack tumor cells and bolster broader immune responses [191,192].

### 2.8. Tumor-Associated Endothelial Cells

Endothelial cells (ECs) are a group of cells that form a cell monolayer lining the luminal side of blood and lymphatic vessels. This barrier controls the passage of various substances, as well as cells and pathogens from the bloodstream, to the surrounding tissues [193]. They are involved in maintaining vessel homeostasis by regulating the following physiological processes: vascular hemodynamics, vascular permeability, coagulation, inflammation, and angiogenesis [193,194]. Under physiological conditions, ECs are typically found in a quiescent form (non-proliferating). However, their activation can rapidly arise in response to pathological conditions such as inflammation and hypoxia, present in the tumor microenvironment, resulting in the tumor-promoting EC phenotype—tumor-associated ECs (TECs) [195]. In comparison to normal ECs, TECs exhibit an altered phenotype in terms of morphology, genetic expression, and function [195,196]. These anomalies result in irregular and leaky tumor blood vessels that contribute to high intratumoral interstitial pressure, tumor hypoxia, and metastasis.

TECs are an essential component of tumor vasculature and play a crucial role in various aspects of tumor growth and progression. By promoting tumor angiogenesis, i.e., the formation of new blood vessels from pre-existing vessels, TECs supply growing tumors with nutrients and oxygen [197,198]. TECs also provide a supportive microenvironment for tumor growth and progression through the secretion of growth factors, cytokines, and extracellular matrix components [199], as well as the differentiation to cancer-associated fibroblasts via endothelial-to-mesenchymal transition (EndMT) [200]. Another TEC activity that supports tumor progression is stimulation of tumor metastasis by providing a route for tumor cells to escape into the bloodstream and colonize distant organs [201]. TECs may also provide signals that actively promote cancer cell metastasis [202] or protect the circulating cancer cells from anoikis by attaching to them via adhesion molecules [203]. Finally, crosstalk between TECs and immune cells can modulate the immune responses within the tumor microenvironment, contributing to immune evasion and tumor progression. TECs may drive immunosuppression through several mechanisms: (1) down-regulation of antigen presentation and recruitment of immune effector cells [204]; (2) induction of expansion of immunosuppressive cell populations, [205] such as Treg cells [206]; (3) inhibition of activation of CD4+ cells and Th1 polarization by TIM-3-expressing TECs [207]; (4) induction of apoptosis of activated anti-cancer CD8+ T cells by FasL-expressing TECs [208]; (5) up-regulation of the immunosuppressive enzyme Indoleamine 2, 3-Dioxygenase 1 (IDO1), which drives T cell apoptosis, inhibition of T cell proliferation, and activation of Treg cells [209]; and (6) up-regulation of PD-L1, which is a negative regulator of T cell activation [210].

Overall, TECs are crucial players in the tumor microenvironment and represent a promising target for anti-cancer therapies. Indeed, currently developed anti-cancer therapeutic strategies combine anti-angiogenic treatments with immunotherapy (bi-specific antibody, immune checkpoint inhibitor, CAR-T cells) in order to modulate both TECs and immune cells for angiogenesis inhibition and the enhanced recruitment and activation of effector cells within the tumor microenvironment [195]. A distinct subset of endothelial cells, known as endothelial progenitor cells, can be readily isolated and genetically modified ex vivo through viral transduction. The therapeutic efficacy of cells expressing thymidine kinase has been demonstrated in a murine model of human ovarian cancer [211]. These cells offer the advantage of selectively targeting tumors and their associated vasculature. There are studies focusing on targeting specific markers expressed by TECs, such as TEM7 and CD276, which have been found in significantly higher levels in tumor-bearing mice. These markers were also observed in human cancer patients, with elevated levels of tumor-associated endothelial cells correlating with the presence of cancer [196,212].

Another type of endothelial cell, known as blood late outgrowth endothelial cells (BOECs), which arise from the prolonged culture of adherent peripheral blood mononuclear cells, can be genetically engineered to produce viral vectors, such as the oncolytic attenuated measles virus of the Edmonston B strain. These carrier cells have demonstrated the ability to deliver and transmit the virus to glioma cells, leading to tumor cell death in murine models [205].

### 2.9. Adipocytes

WAT (white adipose tissue) is histologically a soft connective tissue. WAT is an active endocrine system and can regulate tumor growth, invasion, and metastasis through the production of metabolites, hormones, and cytokines (adipokines) [213]. Normal adipocytes are converted to tumor-associated adipocytes (CAA) by cancer cells [214]. They are characterized by smaller size, phenotypic similarity to fibroblasts, overexpression of collagen VI, and low expression of adiponectin (APN), as well as increased expression of the chemokines CCL2 and CCL5, IL-1β, IL-6, TNF-α, VEGF, and leptin, compared to typical adipocytes. The pro-neoplastic effect of adipocytes is expressed particularly in obese individuals. Adipocytes in individuals with excessive adipose tissue show increased levels of functional activity, leading to increased production of factors associated with inflammation, hypoxia, angiogenesis, and extracellular matrix remodeling [215]. Hypoxic conditions result in the activation of hypoxia-induced factor 1 (HIF-1) in adipocytes, which has been linked to a poor prognosis in obese pediatric cancer patients [216]. Chronic inflammation found in obese individuals also leads to the secretion of elevated levels of cytokines such as IL-6 and TNF-α. Despite their general pro-cancer effect, it was possible to use adipocytes in the fight against cancer. The application of adipocytes in cell-based anticancer therapy leverages their capacity to directly transport anticancer drugs to the tumor site. Adipocytes loaded with a ROS-responsive doxorubicin prodrug and rumenic acid have demonstrated significant anticancer activity in both in vitro and in vivo studies [217]. Although there is some evidence from preclinical studies suggesting that adipocytes may inhibit tumor growth under certain conditions, much of the research remains focused on understanding their dual roles in promoting or inhibiting cancer. Clinical trials still need to investigate the direct application of adipocyte-based therapies in cancer patients to establish their efficacy and safety in real-world settings [218].

The summary regarding discussed cell-based therapies in terms of solid tumor treatment is given in Table 2.

**Table 2:** Cell-based therapies of solid tumors.

Cell Type	Cell Therapy	Description of Cellular Therapy	References
Macrophages	Macrophage-based drug delivery system	The research is the biological phenomenon referred to TRAnsfer of Iron-binding protein (TRAIN). This is based on the direct transfer of the ferritin-drug complex from macrophages to cancer cells. The utilization of macrophages in this context serves as a carrier used for transport of the ferritin-drug complex to hypoxic regions that would otherwise remain inaccessible to alternative therapeutic approaches.	[219,220,221]
	CAR-macrophages for the treatment of HER2-overexpressing solid tumors	Non-replicating adenoviral vectors can effectively deliver CARs to human macrophages. Injection of adenovirus triggers a pro-inflammatory tumor microenvironment (TME) and activates macrophages towards the M1 phenotype. CAR-expressing macrophages transduced with adenovirus can more effectively activate T lymphocytes, significantly prolong the survival curve, and reduce metastasis formation.	[102]
Dendritic cells	Pulsed DC vaccination	Ex vivo DCs are generated from circulating blood precursors or bone marrow progenitor cells. They are educated (pulsed) with the patient’s tumor antigens or tumor-derived mRNA and then introduced back to the patients.	[222,223,224,225,226]
	Hybrid cells vaccination	Induction of tumor-specific CTL by a vaccine made with a fusion of autologous/allogenic DCs and tumor cells extracted from the patient.	[227]
Neutrophils	Neutrophil activation	Ex vivo neutrophil-activating therapy consisting of TNF, anti-CD40, and tumor-binding antibody allows rapid recruitment of neutrophils to tumors.	[163]
	Neutrophil-based drug delivery system	Transport of anticancer drug-loaded nanomaterials by neutrophils.	[163,167,228]
	Neutrophil-based photodynamic therapy platform	Transport of nanoparticles loaded with photodynamic agents or antibodies by neutrophils into cancer.	[168,229,230,231]
	Chimeric antigen receptors neutrophils (CAR-Neu)	Pluripotent stem cells are genetically modified through the integration of CARs and subsequently differentiated into neutrophils with enhanced cytotoxic activity specifically targeting cancer cells.	[232]
T lymphocytes	Chimeric antigen receptors T cells (CAR-T)	Isolation of peripheral T cells through apheresis following transduction with a CAR against tumor antigen. CARs are designed to aid the T cell attachment to the specific proteins on the surface of the cancer cells.	[233,234]
	Tumor-infiltrating lymphocytes therapy (TIL)	Isolation of tumor-infiltrating lymphocytes, selection of lymphocytes with antitumor activity, and their proliferation and injection into patients.	[235,236]
	Cytokine-induced killer (CIK) cells (natural killer-like T cells)	Ex vivo expansion of peripheral blood mononuclear cells with anti-CD3 antibodies, IL-2, and IFN-γ. Cytotoxity of those cells is based on contact of natural killer group 2 member D (NKG2D) with its ligand on tumor cells and the perforin-mediated pathways.	[237]
	Gene-modified T cells expressing novel T cell receptors (TCRs)	Isolation of peripheral T cells through apheresis following transduction with a TCR against tumor antigen.	[71]
	T cell-based drug delivery	Drug-loaded liposomes/multilamellar lipid NPs/lipid-coated polymer nanoparticles.	[238]
B lymphocytes	B cell antigen loading and activation	Ex vivo antigen loading and activation of B lymphocytes, which further enhances T cell activation.	[181,182,183,239]
	Hybrid cell vaccination—B cells	Electric fusion of the patient’s tumor cells with the stimulated autologous/allogenic B cells. Those hybrid cells present tumor-associated antigens and allo-MHC molecules.	[189,240]
Natural killer cells	Allogenic NK cell infusions with IL-2 and chemotherapeutics	Haploidentical, related-donor NK cell infusions along with IL-2 injections and cyclophosphamide/methylprednisolone or fludrabine or cyclophosphamide/fludrabine.	[241]
	Adoptive immunotherapy with NK-92 cells	The NK-92 cell line is derived from NK cell lymphoma. Those highly cytotoxic allogenic cells, after irradiation, are injected into the patient.	[242]
	CAR-NK/CAR-NK-92	NK cells or NK-92 cells transduced with retroviral vectors so that they express a chimeric antigen receptor specific to tumor antigens.	[243,244,245]
Cancer-associated adipocytes	Adipocyte-based drug delivery system	Adipocytes are used to encapsulate anticancer drugs in combination with rumenic acid. The drug is subsequently delivered to cancer cells through the activation of lipid metabolic pathways.	[217]
Endothelial cells	Endothelial progenitor cells (EPCs) vehicles for cancer gene therapy	Ex vivo expansion of EPCs and their transduction with viral vectors so that they express suicide genes or Il-2	[246]
	Endothelial progenitor cells (EPCs) vehicles as oncolytic virus transporters	Ex vivo expansion of EPCs and their modification with viral vectors to produce oncolytic viruses directly in the tumor	[247]

## 3. Summary of the Therapeutic Exploitation of the TME

Targeting the TME has garnered significant attention as a promising cancer treatment strategy due to its critical role in tumor progression and therapy response. This approach includes a range of therapies aimed at various TME components, such as immunotherapies and cell-based therapies. Despite advancements in this field, challenges persist, including the heterogeneity of the TME and its dynamic interactions with cancer cells.

The therapeutic potential of targeting the TME through immune system cells is a key focus in anticancer treatment. Checkpoint inhibitors have revolutionized cancer treatment by blocking proteins that prevent immune cells from attacking cancer. These inhibitors target proteins such as PD-1, PD-L1, and CTLA-4, enhancing the immune system’s ability to fight cancer. By disrupting these checkpoints, immune cells can recognize and destroy cancer cells more effectively. This approach has shown significant success in treating various cancers, including melanoma, lung cancer, and renal cell carcinoma. Despite their efficacy, not all patients respond to checkpoint inhibitors, and research is ongoing to understand and overcome resistance mechanisms [248].

Utilizing macrophages, NK cells, T lymphocytes, and dendritic cells allows for the “reprogramming” of the local immune-suppressive environment of the tumor, thereby enhancing the patient’s immunity [249]. Strategies to reduce immunosuppressive or pro-tumor cells in the TME often involve activating selected immune cell populations. CAR-T cell therapy, initially developed with T lymphocytes, has expanded to include other cells, such as macrophages and NK cells, due to its effectiveness and versatility [7,250].

Vaccination with immune cells has also become an important component of immunotherapy. In 2010, the first vaccines containing dendritic cells were approved for prostate cancer patients. Immune cells used in these therapies can be sourced from the patient’s peripheral blood, stem cells, or genetically engineered cells [112,251]. Incubating specific cell types with cytokines or antibodies can significantly boost their activity, as demonstrated with NK cells [121]. Recently, Tumor-Infiltrating Lymphocyte (TIL) therapy has gained attention with its recent FDA approval. TIL therapy involves isolating lymphocytes from a patient’s tumor, expanding them in the lab, and reinfusing them into the patient. This process aims to boost the body’s immune response directly at the tumor site. TIL therapy has shown promise, particularly in treating melanoma, where it has achieved notable response rates in patients who have not responded to other treatments. The approval of TIL therapy marks a significant milestone in personalized cancer treatment, providing new hope for patients with refractory tumors [252].

The future of cancer immunotherapy lies in the development of integrative and personalized approaches targeting the TME. Therapies targeting or harnessing TME represent the cutting-edge advancement in immuno-oncology, offering novel strategies for treating cancers resistant to traditional therapies. For example, using tumor-infiltrating lymphocytes (TILs), lymphocytes with genetically engineered TCRs, and CAR-T cells holds promise for overcoming resistance mechanisms and enhancing therapeutic outcomes. The development of these therapies highlights the potential of harnessing the host immune system to combat cancer, thereby paving the way for more effective and durable treatment options. Emerging trends emphasize combining current immunotherapies, such as checkpoint inhibitors and CAR-T cell therapies, with strategies that modulate TME components, including tumor-associated macrophages, neutrophils, and endothelial cells. Personalized approaches, leveraging particular omics technologies (or multi-omics approach), could enable the design of therapies tailored to the unique composition of an individual’s TME. Advancements in personalized vaccines—such as B cell vaccines—highlight the potential of tailoring immunotherapies to individual tumor profiles. These strategies, leveraging tumor-specific antigens, can elicit potent and sustained anti-tumor immune responses. Moreover, advancements in nanotechnology and bioengineering could enable the precise delivery of immunomodulatory agents directly to the TME, minimizing off-target effects and optimizing efficacy. The development of cell-based therapies will certainly not remain indifferent to the rapid development of artificial intelligence (AI). Integrating AI with patient data, including genomic, transcriptomic, and proteomic information, can one day revolutionize the personalization of cancer immunotherapy. These integrative strategies, coupled with predictive biomarkers and patient-specific interventions, pave the way for a new era in cancer immunotherapy, focusing on durable and highly individualized treatments.

The authors would like to point out the limitation of the above publication, which is primarily the lack of information regarding transcription factors and signaling pathways that are influenced by the above-mentioned cancer treatment methods.

## Abbreviations

ACT	Adoptive Cellular Therapy
ADAM17	A Disintegrin And Metalloproteinase 17
ADCC	Antibody-Dependent Cellular Cytotoxicity
APC	Antigen-Presenting Cell
APN	Adiponectin
Alox5	Arachidonate 5-Lipoxygenase
bFGF	Basic Fibroblast Growth Factor
Breg	Regulatory B Lymphocyte
CAA	Cancer-Associated Adipocyte
CAF	Cancer-Associated Fibroblast
CAR	Chimeric Antigen Receptor
CAR-M	Chimeric Antigen Receptor Macrophage
CAR-NK	Chimeric Antigen Receptor Natural Killer
CAR-T	Chimeric Antigen Receptor T Cell Therapy
CAR-Neu	Chimeric Antigen Receptors Neutrophils
CCL	C-C Motif Chemokine Ligand
CD	Cluster of Differentiation
CIC	Circulating Immune Complex
CIK	Cytokine-Induced Killer
CPI	Checkpoint Inhibitor
CSF1R	Colony-Stimulating Factor 1 Receptor
CTL	Cytotoxic T Lymphocyte
CTLA-4	Cytotoxic T Lymphocyte Antigen 4
CXCL	C-X-C Motif Chemokine Ligand
CXCR1/CXCR2	CXC Chemokine Receptor 1/2
DAMP	Damage-Associated Molecular Pattern
DC	Dendritic Cell
EC	Endothelial Cell
EGF	Epidermal Growth Factor
EndMT	Endothelial to Mesenchymal Transition
eTreg	Effector Regulatory Cell
FcR	Fc Receptor
FGF	Fibroblast Growth Factor
FDA	Food and Drug Administration
GNR	Gold Nanorod
GM-CSF	Granulocyte-Macrophage Colony-Stimulating Factor
GVHD	Graft-Versus-Host Disease
HER2	Human Epidermal Growth Factor Receptor 2
HGF	Hepatocyte Growth Factor
HIF-1	Hypoxia-Inducible Factor 1
HLA	Human Leukocyte Antigen
IDO	Indoleamine 2,3-Dioxygenase
IDO1	Indoleamine 2,3-Dioxygenase 1
IFN	Interferon
IFNα	Interferon Alpha
IFN-γ	Interferon Gamma
IGF	Insulin-Like Growth Factor
IL	Interleukin
IL-4	Interleukin 4
IL-10	Interleukin 10
IL-12	Interleukin 12
IL-13	Interleukin 13
IL-15	Interleukin 15
IL-18	Interleukin 18
IL-35	Interleukin 35
iNOS	Inducible Nitric Oxide Synthase
LPS	Lipopolysaccharide
MDC	Macrophage–Drug Conjugate
MDSC	Myeloid-Derived Suppressor Cell
MHC	Major Histocompatibility Complex
MMP	Matrix Metalloproteinase
MMP9	Matrix Metalloproteinase 9
Mφ	Macrophage
MPO	Myeloperoxidase
Nφ	Neutrophil
NET	Neutrophil Extracellular Trap
NE	Neutrophil Elastase
NKG2D	Natural Killer Group 2 Member D
NK	Natural Killer
NO	Nitric Oxide
NP	Nanoparticle
OLFM4	Olfactomedin 4
PAMP	Pathogen-Associated Molecular Pattern
PD-1	Programmed Cell Death Protein 1
PD-L1	Programmed Death Ligand 1
PDGF	Platelet-Derived Growth Factor
PGE2	Prostaglandin E2
pDC	Plasmacytoid Dendritic Cell
PlGF	Placental Growth Factor
RNS	Reactive Nitrogen Species
ROBO-1	Roundabout Guidance Receptor 1
ROS	Reactive Oxygen Species
scFv	Single-Chain Variable Fragment
siRNA	Small Interfering RNA
TAM	Tumor-Associated Macrophage
Tc	Cytotoxic T Cell
TCR	T Cell Receptor
TEC	Tumor-Associated Endothelial Cell
Tfh	T Follicular Helper
TGF	Transforming Growth Factor
TGF-β	Transforming Growth Factor Beta
Th	T Helper
TIL	Tumor-Infiltrating Lymphocyte
TME	Tumor Microenvironment
TNF	Tumor Necrosis Factor
TNF-α	Tumor Necrosis Factor Alpha
TRAIL	TNF-Related Apoptosis-Inducing Ligand
TRAIN	TRAnsfer of Iron-binding protein
TRC	T Cell Receptor
TRM	Tissue-Resident Macrophage
Treg	Regulatory T Cell
tTreg	Thymus-Derived Treg Cell
TRUCK	T cells Redirected for Universal Cytokine Killing
VEGF	Vascular Endothelial Growth Factor
WAT	White Adipose Tissue
